# Combination of Medicinal Herbs KIOM-79 Reduces Advanced Glycation End Product Accumulation and the Expression of Inflammatory Factors in the Aorta of Zucker Diabetic Fatty Rats

**DOI:** 10.1155/2011/784136

**Published:** 2011-02-15

**Authors:** Eunjin Sohn, Junghyun Kim, Il Ha Jeong, Chan Sik Kim, Young Sook Kim, Jin Sook Kim

**Affiliations:** Diabetic Complications Research Center, Division of Traditional Korean Medicine (TKM) Integrated Research, Korea Institute of Oriental Medicine (KIOM), 483 Exporo, Yuseong-gu, Daejeon 305-811, Republic of Korea

## Abstract

Previous studies have reported that KIOM-79 shows a strong inhibitory effect on AGE formation and inhibited a proinflammatory state in a murine macrophage cell line. In the present study, we investigated the effect of KIOM-79 on AGE accumulation and vascular inflammation in the aorta of Zucker diabetic fatty (ZDF) rats, a commonly used model of type 2 diabetes. Seven-week-old male ZDF rats were treated with KIOM-79 (50 mg/kg) once a day orally for 13 weeks. We examined the dissected aortas for AGE accumulation, expression of the receptor for AGEs (RAGE), and the expression of proinflammatory factors, including monocyte chemoattractant protein-1 (MCP-1), vascular endothelial growth factor (VEGF), and vascular adhesion molecule-1 (VCAM-1). Nuclear factor-kappaB (NF-*κ*B) and inducible nitric oxide synthase (iNOS) were also measured by Southwestern histochemistry, electrophoretic mobility shift assay (EMSA), and immunohistochemistry, respectively. KIOM-79 markedly reduced the accumulation of AGEs and the expression of RAGE in the aorta. We also found that KIOM-79 attenuated the expression of inflammatory factors including NF-*κ*B, MCP-1, VEGF, VCAM-1, and iNOS in the aortas of ZDF rats. These data suggest that KIOM-79 may prevent or retard the development of inflammation in diabetic vascular disease.

## 1. Introduction

Cardiovascular disease is one of the most common complications of diabetes [1]. Chronic hyperglycemia accelerates the formation of advanced glycation end products (AGEs) and accumulation of AGEs in various tissues. AGE accumulation is an important feature in the development of diabetic macrovascular complications, such as atherosclerosis and cardiac dysfunction, and AGEs are believed to play a crucial role in the pathogenesis of diabetic vascular inflammation [2, 3]. AGE inhibitors, such as LR-90 and aminoguanidine (AG), have anti-inflammatory properties and help protect against diabetic vascular damage [4, 5]. The inhibition of AGE formation, blockade of the AGE-RAGE interaction, and suppression of RAGE expression or its downstream pathways suggest novel therapeutic strategies for the treatment of the vascular complications of diabetes [6, 7]. 

Traditional herbal medicines have been used for the treatment of diabetes or diabetic complications in Korea and Asian countries. The development of KIOM-79, which is a mixture of the 80% ethanol extract of parched Puerariae radix, gingered Magnoliae cortex, Glycyrrhizae rhizome, and Euphorbiae radix, was based on the basic known function of each herb used in traditional Korean medicine for a variety of medical purposes, including diabetes and diabetic complications [8–11]. In our previous studies, KIOM-79 has shown a stronger inhibitory effect on AGE formation than individual herbal medicines in vitro [12]. KIOM-79 has also reduced accumulation of AGEs in the kidney and delayed the development of diabetic nephropathy in animal models for type 1 and 2 diabetes [13–15]. Recent studies have also helped to elucidate the mechanisms of KIOM-79's anti-inflammatory and cytoprotective properties. KIOM-79 blocks both NF-*κ*B and p38 kinase activation in a murine macrophage cell [16] and induces hemoxygenase-1 induction in a pancreas-beta cell line, increasing its antioxidant capacity [17].

These pharmacological actions of KIOM-79 may be useful for the vascular inflammation of the aorta in diabetes. Therefore, the present study was designed to investigate the effects of KIOM-79 on the aortic inflammation in a rat model of type 2 diabetes, the Zucker diabetic fatty (ZDF) rat.

## 2. Methods

### 2.1. Preparation of KIOM-79

The cortex of *Magnolia officinalis* Rehd. et Wils. (Magnoliaceae), radix of *Pueraria lobata* Ohwi (Leguminosae), radix of *Glycyrrhiza uralensis* Fisch (Leguminosae), and radix of *Euphorbia pekinensis* Ruprecht (Euphorbiaceae) were collected from the province Gamsuk in China in 2003 identified by botanist Professor J. H. Kim (Department of Life Science, Kyungwon University, Republic of Korea). All voucher specimens were deposited at the herbarium of the Korea Institute of Oriental Medicine (nos. 1240, 2, 7, and 207, resp.). These medicinal herbs, which constitute KIOM-79, were prepared as previously described in [16]. The quality of KIOM-79 was controlled by HPLC [14].

### 2.2. Animals and Treatments

All animal procedures were carried out in accordance with the National Institute of Health Guide for the Care and Use of Laboratory Animals (NIH publication no. 85-23) and were approved by the Committee on Animal Care of our institution. Male Zucker diabetic fatty (ZDF *fa/fa*) rats and age-matched Zucker lean (ZL *fa*/+ or +/+) rats were used (Charles River Laboratories, USA) at six weeks of age. They were kept in an automatically controlled room (the room temperature was about 24°C and the humidity was about 60%) with a conventional lighting regimen. At 7 weeks of age, they were divided into four groups: (1) Zucker lean rats (ZL, *n* = 7); (2) untreated ZDF rats (ZDF, *n* = 6); (3) ZDF rats treated with 50 mg/kg body weight aminoguanidine (AG, *n* = 6); (4) ZDF rats treated with 50 mg/kg body weight KIOM-79 (KIOM-79, *n* = 7). KIOM-79 and AG were dissolved in water and given orally. Rats were allowed free access to water and food for 13 weeks, including adaptation for one week. At the end of the experimental period, rats were anesthetized with diethyl ether and killed. After the aorta was removed, one part was flash frozen in liquid nitrogen and stored at −80°C until analysis and one part fixed with 4% paraformaldehyde for histological analysis.

### 2.3. Reverse Transcription Polymerase Chain Reaction (RT-PCR) and Real-Time PCR

Total RNA isolation and RT-PCR were as previously described in [13]. Total RNA was then reverse transcribed to cDNA using the IScript cDNA synthesis kit (Bio-Rad, USA). The following sequences were performed for each PCR reaction. Primer sequences used for the analyses of RAGE and *β*-actin mRNA were as follows: RAGE sense, 5′-ACT ACC GAG TCC GAG TCT ACC A-3′; RAGE antisense, 5′-GCT CTG ACC GAA GCG TGA-3′; *β*-actin sense, 5′-TCA TTG ACC TCA ACT ACA-3′; *β*-actin antisense, 5′-CAA AGT TGT CAT GGA TGA CC-3′. PCR products were run on a 1.2% agarose gel containing ethidium bromide (EtBr) and quantitated with densitometry (Las-3000, Fuji photo, Japan). Real-time quantitative PCR analysis was carried out in a 48-well plate using the Opticon MJ Research instrument (Bio-Rad, USA). The following primers were used in this study: MCP-1 sense, 5′-GAG TCG GCT GGA GAA CTA CAA GAG; MCP-1 antisense, 5′-ATG TAC TTC TGG ACC CAT TCC TTA TTG-3′; VEGF sense-CCG TCC TGT GTG CCC CTA ATG-3′; VEGF antisense TCT CTC CTA TGT GCT GGC TTT GG-3′; VCAM-1 sense, 5′-CCC AAA CAAA GGC AGA GTA CAC AG-3′; VCAM-1 antisense, 5′-TTG AGC AGG TCA GGT TCA CAG G-3′; GAPDH sense, 5′-CAA GTT CAA CGG CAC AGT CAA GG-3′; GAPDH antisense, 5′-ACA TAC TCA GCA CCA GCA TCA CC-3′. Real-time PCR was performed in identical conditions. Amplification and quantification of the target gene expression were performed with the iTaq SYBr green mix (Bio-Rad, USA) and Bio-Rad Chromo 4/Opticon system. Relative quantification was performed with the relative comparative threshold (CT) method and analyzed using the software provided by the manufacturer. 

### 2.4. Immunohistochemical Staining

Immunohistochemistry was conducted on paraffin sections using the following antibodies: goat polyclonal anti-MCP-1 and VCAM-1 (Santa Cruz, USA), mouse monoclonal anti-iNOS (BD transduction Lab, USA), and mouse monoclonal anti-AGEs (6D12, Transgenic, Japan). Paraffin-embedded aorta sections (4 *μ*m thick) were deparaffinized, rehydrated, and treated with 3% H_2_O_2_ for 10 min. Sections were blocked with 2% animal-free serum for 30 min at 37°C. Following 1-hour incubation with primary antibodies, sections were incubated with an LSAB kit (DAKO, USA) and visualized by 3,3′-diaminobenzidine tetrahydrochloride or nitroblue tetrazolium (NBT) and 5-bromo-4-chloro-3-indolylphosphate (BCIP). The sections were counterstained with hematoxylin. All stained sections were visualized on a computer display with an Olympus DP71 camera connected to a light Olympus microscope (Tokyo, Japan). The degree of protein expression was analyzed using Image J software (NIH) by an observer blinded to the experimental design. 

### 2.5. Western Blot Analysis

The aortas from each group (0.1–0.2 g) were lysed in solutions containing 250 mM sucrose, 1 mM ethylenediaminetetraacetic acid (EDTA), 0.1 mM phenylmethylsulfonyl fluoride (PMSF), and 20 mM potassium phosphate buffer, at pH 7.6 with a homogenizer at 3000 rpm. The protein was separated to PVDF membranes (Bio-Rad, USA). Membranes were probed with anti-RAGE and iNOS antibody (BD transduction Lab, USA). The bound horseradish peroxidase-conjugated secondary antibody was detected using an enhanced chemiluminescence detection system (iNtRON Biotechnology, Republic of Korea). Protein expression levels were determined by analyzing the signals captured on the PVDF membranes using an image analyzer (Las-3000, Fuji photo Japan).

### 2.6. Measuring of NF-*κ*B Activity

To localize the NF-*κ*B activity in aorta section, Southwestern histochemistry was performed as described by Koji et al. [18]. Briefly, complementary oligonucleotides containing an NF-*κ*B binding consensus sequence were synthesized as follows: 5′-AGT TGA GGG GAC TTT CCC AGG C-3′. The probe was labeled with digoxigenin (Roche, Germany). Formalin-fixed and paraffin-embedded aorta tissue sections were deparaffinized and rehydrated. Sections were subsequently digested with pepsin A (433 U/mg) and then incubated with the labeled probe (100 pM) at 37°C overnight. After washing, sections were incubated with antidigoxigenin antibody conjugated with alkaline phosphatase (Roche, Germany) for 1 hour at 37°C. The color reaction was developed using NBT/BCIP. The following were used as negative controls: (1) absence of probe, (2) mutant NF-*κ*B probe labeled with digoxigenin, and (3) competition assays with a 200-fold excess of unlabeled NF-*κ*B followed by incubation with labeled probe. In each case, the numbers of positive cells in the aorta section were counted in a blinded manner. For EMSA assay, nuclear extracts from the whole aorta were prepared with a kit according to the manufacturer's instructions (Pierce Biotechnology, USA). EMSA assay was performed by incubating 15 *μ*g of nuclear protein extract with IRDye 700-labeled NF-*κ*B oligonucleotide (LI-COR bioscience, USA) or unlabelled probe for cold competition. EMSA gels were analyzed and images were captured and quantified using the LI-COR Odyssey infrared laser imaging system (LI-COR Bioscience, USA).

### 2.7. Data Analysis

For multiple experiments, the results are expressed as mean ± S.E.M. Two-tailed Student's *t*-tests were used to compare two groups using PRISM software (Graph Pad, USA). Values of *P* < .05 were considered statistically significant.

## 3. Results

### 3.1. Accumulation of AGEs in the Aorta

**Figure 1:**
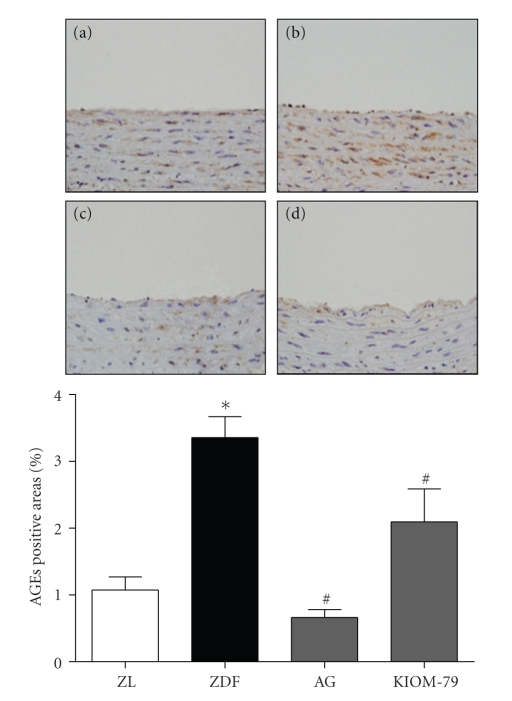
The effects of KIOM-79 treatment on the expression of AGEs in the aorta of ZDF rats. This figure included representative histological staining of the aorta. AGE staining in the aorta of ZL rats (a), untreated ZDF rats (b), ZDF rats treated with AG (50 mg/kg) (c), and ZDF rats treated with KIOM-79 (50 mg/kg) (d). Original magnification: ×400. Quantitative analysis of the AGE stain was calculated. All data were expressed as mean ± S.E.M. *P < .01 compared with ZL rats; ^#^ *P* < .01 compared with untreated ZDF rats.

We investigated the effect of KIOM-79 on AGE accumulation in the aortas of ZDF rats using immunohistochemical staining. Using quantitative analysis, we determined that the expression of AGEs in untreated ZDF rat was increased compared with age-matched ZL rats. The treatment of KIOM-79 or AG in ZDF rats significantly reduced the AGE expression by 37.8 and 80.4%, respectively, compared to untreated ZDF rats (*P* < .01) (Figure 1).

### 3.2. Expression of RAGE in the Aorta

**Figure 2:**
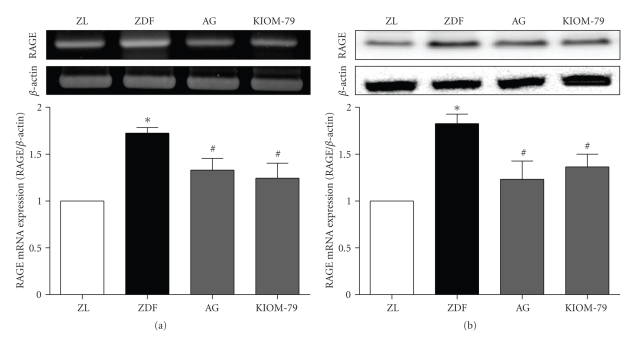
The effects of KIOM-79 treatment on the expression of RAGE in the aorta. The relative levels of specific mRNAs (a) and protein (b) were assessed by RT-PCR and Western blot analysis. Results were normalized to actin. All data are expressed as mean ± S.E.M. *P < .01 compared with ZL rats; ^#^ *P* < .05 compared with untreated ZDF rats.

Inhibition of the AGEs/RAGE interaction prevents the progression of the pathogenetic pathway for diabetic complications [19]. Thus, to determine whether KIOM-79 inhibits RAGE expression in the aorta of diabetic rats, RAGE expression was determined by RT-PCR and Western blot. As shown in Figures 2(a) and 2(b), RAGE mRNA and protein expression in the untreated ZDF rats was significantly increased compared with ZL rats, whereas KIOM-79 or AG markedly decreased its expression compared with untreated ZDF rats (*P <* .05).

### 3.3. Expression of Inflammatory Factors in the Aorta

**Figure 3:**
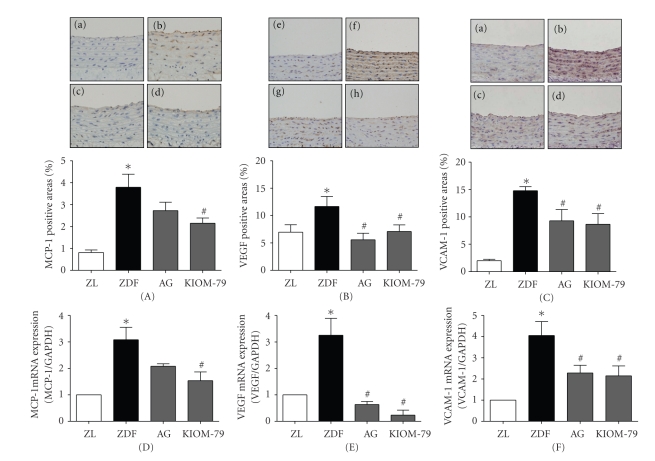
The effects of KIOM-79 treatment on expression of MCP-1, VEGF, and VCAM-1 in the aorta. Representative immunohistochemistry images for MCP-1 (A), VEGF-1 (B), and VCAM-1 (C) staining in the aorta of Zucker lean rats ((a), (e), and (i)), untreated ZDF rats ((b), (f), and (j)), ZDF rats treated with AG (50 mg/kg) ((c), (g), and (k)), and ZDF rats treated with KIOM-79 (50 mg/kg) ((d), (h), and (l)). Original magnification: ×400. After real time-PCR analysis, MCP-1 (D), VEGF (E), and VCAM-1 (F) levels were normalized to GAPDH. All data are expressed as mean ± S.E.M. *P < .01 compared with ZL rats; ^#^ *P* < .05 compared with untreated ZDF rats.

We examined the effect of KIOM-79 on the expression of MCP-1, VEGF, and VCAM-1, all of which are contributed to vascular inflammation. We measured the expression of MCP-1, VCAM-1, and VEGF in the aorta by immunohistochemistry and found that MCP-1, VEGF, and VCAM-1 were expressed throughout the aorta of untreated ZDF rats compared with ZL rats. ZDF rats treated with KIOM-79 or AG had significantly reduced levels of MCP-1, VEGF, and VCAM-1 expression compared with untreated ZDF rats (*P* < .05) (Figures 3(A), 3(B), and 3(C)). However, AG-treated ZDF rats had only a slight decrease in MCP-1 staining. To corroborate our immunohistochemical findings, we measured the mRNA expression of MCP-1, VEGF, and VCAM-1 by real-time-PCR. As shown in Figures 3(D)–3(F), the mRNA levels of MCP-1, VEGF, and VCAM-1 in untreated ZDF rats were significantly increased compared with ZL rats. Treatment with KIOM-79 or AG markedly decreased these inflammatory factors compared to untreated ZDF rats (*P* < .05). ZDF rats treated with AG showed a slight reduction of MCP-1 mRNA expression and a significant decrease in the mRNA level of both VEGF and VCAM-1 (*P* < .05).

### 3.4. Activation of NF-*κ*B and Expression of iNOS in the Aorta

**Figure 4:**
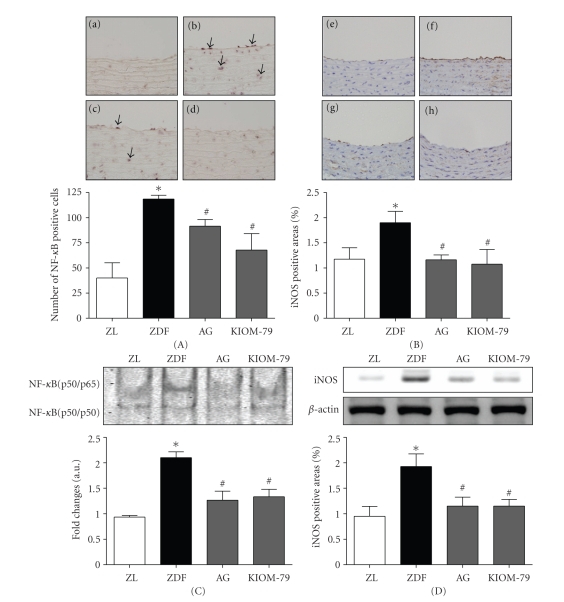
The effects of KIOM-79 treatment on activation of NF-*κ*B and iNOS in the aorta. Representative NF-*κ*B (A) and iNOS (B) staining in the aorta in Zucker lean rats ((a), (e)), untreated ZDF rats ((b), (f)), ZDF rats treated with AG (50 mg/kg) ((c), (g)), and ZDF rats treated with KIOM-79 (50 mg/kg) ((d), (h)). NF-*κ*B staining was observed in the nuclei of both the endothelial layer and smooth muscle lesion (arrow), and iNOS staining (brown color) was observed in the aorta. Hematoxylin counterstain. Original magnification: ×400. Analysis of EMSA of NF-*κ*B activation (C) and Western blot of iNOS (D). Quantitative analyses of the NF-*κ*B and iNOS stains were completed. All data were expressed as mean ± S.E.M. *P < .01 compared with ZL rats; ^#^ *P* < .05 compared with untreated ZDF rats.

We then investigated whether KIOM-79 inhibits the NF-*κ*B activity by using Southwestern histochemistry. This method allowed the localization of the activated nuclear factor in aorta. Untreated ZDF rats had markedly increased NF-*κ*B positive staining in the aorta section (both endothelial layer and smooth muscles lesion) compared to ZL rats (approximately 290.2% of ZL rats) (Figure 4(A)a–d). Treatment with KIOM-79 or AG significantly decreased NF-*κ*B staining in the aorta by 42.2 or 22.8% compared to untreated ZDF rats. We also confirmed that nuclear extracts from the whole aortic tissues were subjected to analysis for NF-*κ*B DNA-binding activity as measured by EMSA. The results showed that NF-*κ*B DNA-binding activity was significantly reduced in treated with KIOM-79 or AG in ZDF rats (Figure 4(C)). Quantitative analysis showed an increased number of NF-*κ*B positive staining and NF-*κ*B activity in aorta section of ZDF rats compared with ZL rats. KIOM-79 or AG treatment significantly reduced the NF-*κ*B staining and activity in the ZDF rats (*P* < .05). 

We examined the expression of iNOS in the aorta by immuohistochemistry and Western blot analysis. There was a striking difference between the amount of iNOS present in the ZL rats and that in untreated ZDF rats (approximately 162% of ZL rats) (Figure 4(B)e–h). iNOS staining was significantly decreased in ZDF rats treated with either AG or KIOM-79 by 38.9 or 43.7%, respectively, compared to untreated ZDF rats (*P* < .05). The expression of iNOS was markedly increased in the untreated ZDF rats (Figure 4(D)). However, the treatment of KIOM-79 or AG significantly decreased the expression of iNOS.

## 4. Discussion

Several studies have demonstrated that vascular inflammatory processes play a crucial role in the development of diabetic macrovascular dysfunction [20]. In the present study, in order to verify the preventive effects of KIOM-79 in diabetic macrovascular disease, we investigated whether the administration of KIOM-79 inhibited the expression of proinflammatory cytokines and chemokines in the vascular tissue of Zucker diabetic fatty (ZDF) rats. In our previous study, the treatment of KIOM-79 in STZ-induced diabetic rats (250 and 500 mg/kg body weight) has dose-dependently prevented the development of diabetic nephropathy [14]. KIOM-79 (500 mg/kg body weight) retarded the development of diabetic nephropathy in Goto-Kakizaki rats [15]. Diabetic db/db mice treated with KIOM-79 (150 mg/kg body weight) show reduced apoptotic cell death and accumulation of AGEs in the retina [21]. Recently, it was reported that the long-term treatment of KIOM-79 (50 mg/kg body weight) in ZDF rat prevented the progress of diabetic nephropathy [22]. Based on our previous studies, we assumed that the minimum effective dose of KIOM-79 is 50 mg/kg body weight in animal models. Thus, in this study, we investigated the effect of KIOM-79 (50 mg/kg body weight) on diabetic vascular inflammation in ZDF rat, which is a murine model for type 2 diabetes. ZDF rats show the vascular dysfunction seen in the coronary and mesenteric arteries and preceding the changes in conduit (aorta) arteries [23]. In this study, we found that KIOM-79 inhibited the accumulation of AGEs and RAGE expression in the aorta of ZDF rats. Furthermore, KIOM-79 treatment suppressed the diabetic vascular inflammatory process through NF-*κ*B activation, leading to decreased MCP-1, VEGF, VCAM-1, and iNOS.

AGEs, sugar-derived irreversible protein modifications, have been implicated in the pathogenesis of diabetic vascular complications [2, 3]. AGEs exert their cellular effects mainly through interaction with cell-surface receptors, that is, RAGE [6]. The interaction of AGEs and RAGE leads to the activation of the inflammatory response, including the production of cytokines and chemokines. Thus, the inhibition of AGE formation, blockade of the AGE-RAGE interaction, and suppression of RAGE expression or its downstream pathways suggest novel therapeutic strategies for the treatment of the vascular complications of diabetes [6, 7]. AGE inhibitors, such as AG, pyridoxamine, and ALT-711 (alagebrium), have been reported to attenuate numerous functional and structural manifestations of diabetic vascular disease in experimental animals [5, 24]. AG also inhibited the gene expression of the renal and vascular RAGE in a diabetic animal model [25]. Our previous studies have shown that KIOM-79 inhibits the formation of AGEs more *in vitro* than AG and also reduces AGE accumulation in the kidney and retina of type 1 and 2 diabetic animal models. Furthermore, *in vitro* KIOM-79 inhibited collagen-AGE cross-linking better than AG [22]. As our study demonstrated, KIOM-79 also reduced AGE accumulation and RAGE expression in the aorta of ZDF rats (Figures 1 and 2). Our results suggested that KIOM-79 inhibits the accumulation of AGEs in the vascular tissue of ZDF rats. 

NF-*κ*B is a multiprotein complex that can activate various genes involved in multiple cellular functions. In unstimulated cells, NF-*κ*B resides in the cytoplasm in an inactive complex with an inhibitor, kappaB (I*κ*B). Pathogenic stimuli causes the release of I*κ*B and allows NF-*κ*B to enter the nucleus, bind to DNA recognition sites in regulatory regions of the target gene, and influence the transcription of specific genes [26, 27]. Recently, AGEs have been shown to stimulate the activation of NF-*κ*B through binding to RAGE [28]. In the development of the cardiovascular complications of diabetes, AGE-RAGE interaction upregulates the transcription factor NF-*κ*B and its target genes, including the adhesion molecules (VCAM-1 and MCP-1) [4, 29]. Inflammatory factors such as MCP-1 and VCAM-1 accelerate atherosclerosis in diabetic patients [30, 31]. A study by Renier et al. [32] demonstrated that AGEs induced the expression of MCP-1 in endothelial cells and MCP-1 secretion by intima cells attracted monocytes to VCAM-1 on the vessel, leading to an increased inflammatory response. Angiogenic factors, such as VEGF, are also involved in the pathology of chronic inflammatory disease [33]. AGEs have been shown to induce VEGF expression in retinal endothelial cells, and a direct stimulatory effect of AGEs on retina vascular VEGF expression has been documented [34]. Moreover, NF-*κ*B controls the expression of VEGF both directly [35] and indirectly through the induction of expression of AP-1 [36]. Several studies have demonstrated that in vascular smooth muscle cells, NF-*κ*B binds to the iNOS promoter, increasing iNOS expression [37], and iNOS upregulation has been found in microvessels of experimental diabetic rodents [38]. Expression of iNOS in the vessel may contribute to altered vascular function during inflammation and cardiovascular diseases [39]. Our present study demonstrated that ZDF rats had more activated NF-*κ*B and iNOS, as well as increased MCP-1, VEGF, and VCAM-1 expression, compared to ZL rats, and treatment with KIOM-79 or AG reversed this effect (Figures 3 and 4). AG, the positive control drug, significantly reduced iNOS expression in ZDF rats, as the iNOS-selective inhibitors [37] VCAM-1 gene expression was decreased by AG treatment, consistent with previous findings that AG lowered AGE levels by inhibiting VCAM-1 expression in human vascular endothelial cells exposed to glycated protein [40]. KIOM-79 prevented the upregulation of iNOS and activation of NF-*κ*B in the aorta of diabetic rats, consistent with the recent report that KIOM-79 has exhibited anti-inflammatory effects by blocking NF-*κ*B activation in LPS-induced iNOS gene expression in macrophages [16]. These data suggest that KIOM-79 can protect diabetic vascular inflammation by inhibiting NF-*κ*B activation, which in turn decreases iNOS, VEGF, MCP-1, and VCAM-1 expressions. 

Taken together, we concluded that KIOM-79 inhibits AGE accumulation and reduces RAGE expression in the diabetic aorta. Decreased AGE/RAGE interaction decreases NF-*κ*B activation, subsequently decreasing the expression of multiple inflammatory factors, including MCP-1, VEGF, VCAM-1, and iNOS. The present study shows that KIOM-79 may help in the prevention and delay of diabetes-induced vascular inflammation.
